# Rapamycin reduces peritendinous fibrosis but has a limited effect on intratendinous healing in a rodent Achilles tendon injury model

**DOI:** 10.1038/s41598-026-45606-x

**Published:** 2026-03-26

**Authors:** Neil Marr, Danae E. Zamboulis, Ross E. Beaumont, Zofia J. Tatarczyk, Marianne Pultar, Yu-Mei Chang, Richard J. Piercy, Richard Meeson, Mandy J. Peffers, John C. W. Hildyard, Chavaunne T. Thorpe

**Affiliations:** 1 Comparative Biomedical Sciences, Royal Veterinary College, Royal College Street, London, NW1 0TU UK; 2 Department of Clinical Sciences, School of Veterinary Medicine, Aristotle University of Thessaloniki, Thessaloniki, 54124 Greece; 3 Clinical Sciences and Services, Royal Veterinary College, Hawkshead Lane, Hatfield, AL9 7TA UK; 4 TAmiRNA GmbH, Vienna, Austria; 5 Institute of Life Course and Medical Sciences, University of Liverpool, Apex Building, 6 West Derby Street, Liverpool, L7 9TX UK

**Keywords:** Tendon, Interfascicular matrix, Rapamycin, mTOR, Achilles tendon, Tendinopathy, Tendon healing, Cell biology, Diseases, Medical research

## Abstract

Tendon injuries are common, affecting both athletes and the general population. Healing is often poor, with deposition of fibrotic, disorganised scar-like tissue leading to continued pain, dysfunction and reinjury. The pathogeneses of many fibrotic diseases, including tendinopathies, are influenced by the mammalian target of rapamycin (mTOR) signalling pathway, which modulates processes required for cell growth and proliferation. Rapamycin, an mTOR inhibitor, is a promising therapeutic for several fibrotic diseases, and can facilitate musculoskeletal tissue repair. Therefore, we used a needle-induced injury model of Achilles tendon repair in Wistar rats (*Rattus norvegicus*) to test the hypothesis that rapamycin treatment during the early stages of tendon injury enhances tendon healing via the modulation of resident tendon cell populations. Results demonstrated that, while rapamycin treatment decreased peritendinous fibrosis and appeared to modulate cell recruitment as well as circulating levels of several microRNAs, it did not enhance healing of lesions within the tendon core, up to three weeks post-injury. Consequently, rapamycin is not an effective therapeutic for acute tendon injury in this rodent model. Future studies should establish if rapamycin is able to improve tendon healing in aged animals or with longer-term administration.

**Supplementary Information:**

The online version contains supplementary material available at 10.1038/s41598-026-45606-x.

## Background

Tendons are an essential component of the musculoskeletal system, permitting the transfer of muscle forces across the joint and enabling locomotion. Tendon injury is common, with injuries and ruptures affecting ~ 150,000 people in the UK, and 17 million people worldwide each year^1,2^ with a cumulative Achilles tendinopathy incidence of 6% in the general population, and up to 50% in athletes^3^. Tendon injury is characterised by extracellular matrix (ECM) disruption and cellular infiltration, often accompanied by rupture of collagen fibres in the tendon core^4–6^. Healing is generally poor, with deposition of functionally inferior fibrotic scar tissue often leading to chronic tendinopathy, continued pain and dysfunction^7^. Current treatment is often reliant on non-invasive pain management, rehabilitative methods or use of injectables which have variable effects^8^. Surgical intervention remains part of the mainline treatment approach for tendon injury, yet despite relatively few post-operative complications, there is substantial heterogeneity in outcomes and subsequent requirements for rehabilitation and physical therapy^9–11^. Regenerative therapies to limit local fibrosis and restore tendon function post-injury would consequently be highly beneficial.

Tendons are composed of collagenous fascicles, bound together by the interfascicular matrix (IFM, also referred to as the endotenon), which has diverse roles in tendon homeostasis that are critical to energy-storing tendons such as the human Achilles tendon and equine superficial digital flexor tendon^12–14^. Previous studies have established that the IFM houses several cell populations, and we have reported the specific recruitment of IFM-localised CD146+ (MCAM) cell populations to sites of injury^13,15,16^. This was accompanied by colocalised increases in laminin-α4, an extracellular ligand for CD146^17^, which has well-established roles in wound healing^18^. Though often reported as a tendon stem/progenitor cell population^19,20^, CD146+ cells are implicated in endogenous reparative processes particularly as part of the tendon vascular niche^15,16^, but alone they are insufficient for complete repair with failed healing leading to excess fibrosis, characterised by aberrant cell proliferation and type III collagen deposition. Application of small molecules that enhance the intrinsic repair mechanisms of tendon cell populations is an attractive strategy, and therefore pre-clinical studies are required to identify drug candidates (or druggable targets) that augment tendon healing.

The mammalian target of rapamycin (mTOR) pathway modulates processes required for cell growth and proliferation, including gene and protein synthesis, energy metabolism and autophagy^21^. One mechanism by which the mTOR pathway exerts its functions is via microRNA (miR) regulation^22^. miRs are small, non-coding RNAs (ncRNAs) that regulate gene expression and are packaged in extracellular vesicles (EVs); small membrane bound structures secreted by cells that play important roles in intercellular communication via nucleic acid and protein cargoes^23^. While comparatively little is known about the role of EVs and EV-associated miRs in tendon injury, we have previously identified several circulating miRs associated with injury^16^, and several mTOR-regulated miRs with anti-fibrotic and autophagy promoting functions are altered in tendon and skeletal disease^24–28^. Moreover, the increasing evidence of a vascular niche and associated cell populations within the tendon IFM^15^ imply a high level of communication between blood and tendon environments. Circulating EVs and their cargoes could serve as a surrogate for monitoring the systemic response to tendon injury via an initial angiogenic activation and subsequent processes resulting in tendon inflammation. Given that acquisition of serum in patients is relatively non-invasive, serum-derived EV biomarkers remain an attractive prospect for potential tendon disease diagnostics and therapies.

Rapamycin (sirolimus) is a direct inhibitor of the kinase mTOR through complex formation with its obligatory accessory partner FK506-binding protein 12 (FKBP12) as well as other members of the FKBP family^29^. Rapamycin is a promising therapeutic to treat several fibrotic diseases^30–32^: it facilitates musculoskeletal tissue repair, improving fracture healing and reducing the severity of peritendinous adhesions by inducing autophagy^33–35^. Studies have demonstrated potentially favourable effects when tendon cell populations are treated with rapamycin in vitro^36–38^, though in vivo studies have primarily investigated the anti-ageing benefits of rapamycin treatment^39,40^, or the capacity to potentiate repair under traumatic injury scenarios^41^. Inhibition of the mTOR pathway during the acute phases of tendon injury may therefore limit the progression of fibrosis and improve healing during subsequent stages of tendon healing, with circulating EV cargoes such as miRs potentially contributing towards tendon injury and inflammation at a systemic level. Therefore, this focused study tested the hypothesis that daily systemic administration of rapamycin during the early stages of an acute, needle-puncture tendon injury modulates resident tendon cell populations and enhances tendon healing. Furthermore, we aimed to assess whether rapamycin administration has a systemic effect in response to tendon injury via modulation of circulating EV-associated miRNAs to identify potential biomarkers associated with rapamycin treatment.

## Materials & methods

### Ethical statement

All procedures described were performed in compliance with the Animals (Scientific Procedures) Act 1986, approved by the Royal Veterinary College Animal Welfare and Ethical Review Board (ID:2016–0096 N; June 2017), conducted under Home Office project license PB78F43EE (license holder: CTT), and are reported according to the ARRIVE guidelines^16,42,43^.

### Housing & husbandry

Female Wistar rats (*n* = 24 total; 12 weeks old; mean weight 206 g (range 141–226 g)) were housed in groups of 3 in individually ventilated polypropylene cages, subjected to 12 h:12 h LD cycles between 08:00 and 20:00 at a temperature of 21℃. Animals were fed *ad libitum* on a maintenance diet (Special Diet Services, Chelmsford, UK) and provided with a rotational enrichment programme.

### Experimental design and surgical procedures

Surgical procedures (**Supplementary Figure ****S1**) were performed as described previously using the left hindlimb for needle-induced tendon injury^16^. Animals were anaesthetised as follows: induction (2% isoflurane; flow rate 2 L/min) was performed using an anaesthetic induction chamber attached to an isoflurane machine with vaporiser, oxygen concentrator, and Fluovac active scavenger unit (Vet-Tech Solutions Ltd., Cheshire, UK). Animals were given pre-operative analgesia (0.05 mg/kg buprenorphine, sub-cutaneous route) and placed in prone position on a heat pad for surgery. Anaesthesia was maintained via isoflurane inhalation (3%; flow rate 2 L/min) delivered using a nose-cone veterinary mask (Vet-Tech Solutions Ltd., Cheshire, UK). Achilles tendons were punctured 2 mm proximal to the calcaneal insertion using a 21G sterile hypodermic needle by passing the needle through the tendon once. Non-operated contralateral hindlimbs were used as controls (we have previously shown there is no difference in cell or extracellular matrix between sham-operated and non-operated tendons^16)^. All surgeries were performed by a single operator (RM). Rapamycin (powder; R-5000; LC laboratories, Massachusetts, USA) was dissolved in dimethyl sulfoxide (DMSO) at 100 mg/ml and diluted to a final concentration of 1 mg/ml in 5% (w/v) polyethylene glycol (PEG; P3265; Sigma-Aldrich, Poole, UK) and 5% (v/v) TWEEN 80 (P1754; Sigma-Aldrich, Poole, UK) in sterile-filtered water (W3500; Sigma-Aldrich, Poole, UK). Rapamycin solution was sterile-filtered and stored at − 80 °C until use. Vehicle solution consisted of 5% PEG and 5% TWEEN 80 in sterile water. Animals were administered analgesia for 48 h post-surgery (total doses = 5; 0.05 mg/kg buprenorphine, sub-cutaneous). Gait was monitored for potentially adverse effects due to surgery, and weight (severity threshold = 20% weight loss) was monitored for adverse reaction to rapamycin treatment. No adverse gait or weight loss was observed throughout the entire study.

**Fig. 1:**
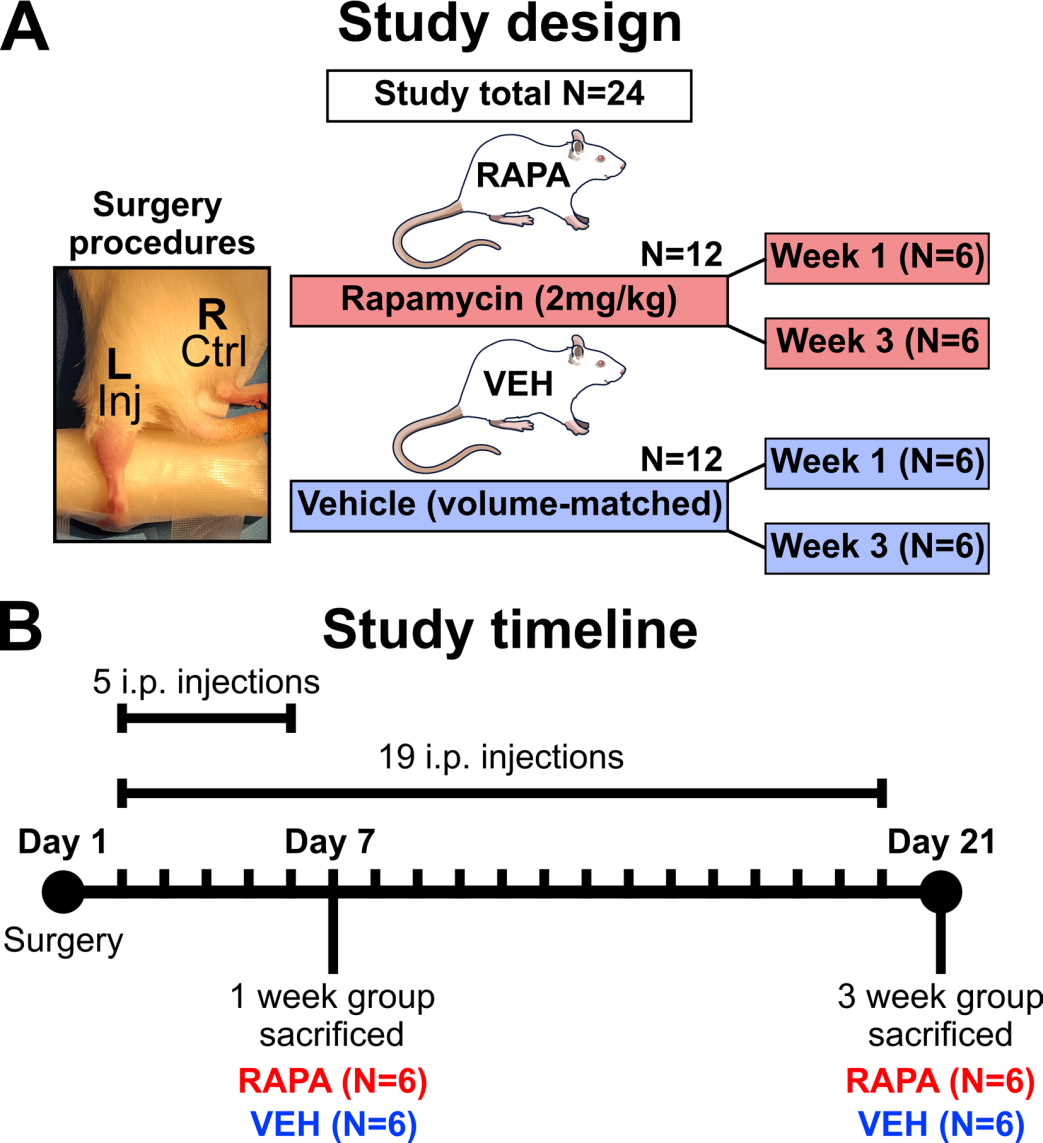
Experimental design and timeline. **(A)** Rats were subjected to needle-induced Achilles tendon injury in the left hindlimb, with the contralateral right intact tendon acting as the control. A total of 24 rats was used and grouped by treatment with rapamycin (RAPA) or volume-matched vehicle (VEH), and sub-grouped by timepoints at 1 week and 3 weeks. **(B)** Study timeline began at day 1 with surgery performed, followed by daily i.p. injections until the day preceding sacrifice. For treatment regimes, 1-week subgroups received their first dose at day 2 and final dose at day 6 (five total injections), and week 3 subgroups similarly were dosed from day 2 until receiving their final injection on day 20 (19 total injections). Rat schematic adapted from NIAID Visual Medical and Arts. 10/01/2025. Black rat. NIAID NIH BIOART Source. bioart.niaid.nih.gov/bioart/54.

Experimental design and procedures are outlined in Fig. 1. Animals were allocated to groups using a randomisation sequence (4 groups: treated/untreated, collected at either day 7 or day 21, *n* = 6/group). All groups underwent surgery to their left Achilles tendon to induce injury. Treated animals were injected daily (intraperitoneal; i.p.) with 2 mg/kg rapamycin (RAPA), while untreated animals received volume-matched vehicle (VEH) solution (Fig. 1A). Dosing regimens (Fig. 1B) were started 24 h post-surgery and ended 24 h prior to euthanasia. For the day 7 groups, animals consequently received a total of five i.p. injections, whereas day 21 groups received a total of 19. Euthanasia was conducted via rising CO_2_ inhalation (chamber fill rate: 20% volume/min) and confirmed with cardiac puncture (blood collection) and cervical dislocation.

### Tissue & serum processing

Left and right Achilles tendons were harvested from cadavers within 2 h postmortem. Tendons were snap-frozen (dry ice-chilled hexane) in optimal cutting temperature embedding matrix (OCT; Cell Path, Newtown, UK) then stored at − 80 °C. Cadavers were examined post-mortem for abnormalities in major organs including liver, heart, lungs, and kidneys. No gross pathologies were observed. Blood samples were collected into plain tubes following cardiac puncture, allowed to clot for 30 min, then spun at 1500 RCF for 10 min and serum harvested. All samples were stored at − 80 °C prior to analysis.

### Histology

Coronal serial sections (12 μm) were prepared from OCT-embedded tendons, mounted on glass slides (Epredia SuperFrost Plus) with ~ 3 sections mounted per slide and air-dried at room temperature (RT) for 2 h before storage at − 80 °C. Thawed slides (two slides per sample; *n* = 6 per treatment group/timepoint) were fixed in methanol: acetone (1:1; 5 min.), washed with dH_2_O, and haematoxylin and eosin (H&E) stained as described previously^16^. Sections were imaged using a Zeiss Axioscan slide scanner (×20 objective, Zeiss, Oberkochen, Germany). Sections with obvious mounting artifacts (tears, folds or inadequate staining) were excluded from analysis. Images were scored as performed previously^16^ by two independent blinded scorers (RB & DZ), using a modified Bonar scoring system^44^ to assess cellularity, cell morphology, tissue organisation, and vascularity (see **Supplementary Table 1**). Scoring also included a fifth category which assessed the peritenon independently, given previous studies reporting that rapamycin enhances peritenon healing^35^. Scores in each category were summed to give a total score. Inter-observer variability was assessed by calculating linear weighted Kappa statistics^45^ using an online software tool (https://vassarstats.net/kappa.html).

### Immunohistochemistry

Immunohistochemistry was performed as previously described^16^. Fixed sections (three sections per slide; four slides per sample; *n* = 6 per treatment group/timepoint) were washed with Tris-buffered saline (TBS) and incubated with blocking buffer (5% rat serum and 5% goat serum diluted in TBS containing 1% bovine serum albumin (BSA)) for 2 h at room temperature (RT). Sections were incubated with primary antibodies (anti-CD146, Abcam ab75769, 1:100; anti-Collagen III, Abcam ab6310, 1:100) diluted in blocking buffer (described above) overnight at 4°C. Sections were washed with TBS, treated with 0.3% hydrogen peroxide for 15 mins, and incubated with secondary antibodies (goat anti-rabbit IgG, Dako; goat anti-mouse IgG, Dako, both at 1:500) diluted in blocking buffer for 1 h at RT. Sections were developed for 5 mins with 3,3’-diaminobenzidine (DAB, Vector Labs, San Francisco, CA, USA), counterstained with haematoxylin for 30 s, dehydrated, and mounted with DPX (Sigma-Aldrich, Poole, UK). Sections were cured overnight and imaged using a slide scanner and ×20 objective (Zeiss Axioscan, Oberkochen, Germany).

### Quantitative reverse transcription PCR (RT-qPCR)

Quantitative reverse transcription PCR (RT-qPCR) was performed as previously described^43^. Briefly, RNA was isolated from cryosections of OCT-embedded tendons (30–50 cryosections, *n* = 6 per treatment group/timepoint), collected serial to those mounted for histology. RNA was isolated using TRIzol as per manufacturer’s guidelines with an additional 1:1 chloroform extraction following phase separation. Purity of RNA was measured using a spectrophotometer (DS-11; DeNovix), and samples with 260/230 absorbance ratios < 1.7 were re-precipitated using isopropanol. Samples that did not pass RNA quality control after this additional step were excluded (a total of 8 samples, from the following treatment groups/timepoints: Veh d7 and d21, Rapa d7 and day 21). cDNA synthesis (800 ng RNA) was performed with a High-Capacity cDNA synthesis kit (ThermoFisher). cDNA was diluted 1:10 with nuclease-free water and stored at − 20 °C. qPCR was performed using a CFX384 thermal (384-well plates; Hard-Shell thin wall, skirted, #HSP3805, BioRad) with PrecisionPLUS SYBR green mastermix (Primerdesign). PCR reactions (10 µL; triplicates) were carried out using 2 µL cDNA per well (ca. 8 ng cDNA). All primers (Table 1) were either selected from literature or designed in-house using Primer3 (exon spanning by default)^46^. All runs included melt curve analysis as standard and template-free controls to confirm single amplicons and primer specificity with amplification. Quantification cycle (Cq) values were determined by regression and converted to Relative Quantities (RQ). The expression of genes of interest (GOI) was normalised to the mean expression of three reference genes (*Actb*, *Hprt1* and *Csnk2a2*) previously determined to be the most stably expressed in rat Achilles tendon^43^.

**Table 1:** Primer sequences used for qPCRs.

Gene symbol	Gene name	Primer sequence (5’−3’)	Reference
*Actb*	Actin Beta	**Forward:** ATTGCTGACAGGATGCAGAA **Reverse**: TAGAGCCACCAATCCACACAG	^47^
*Acta2*	Actin Alpha 2	**Forward**: GCCAGTCGCCATCAGGAAC **Reverse**: CACACCAGAGCTGTGCTGTCTT	^48^
*Col3a1*	Collagen Type III Alpha 1 Chain	**Forward**: GAGGAATGGGTGGCTATCCT **Reverse**: GGTATCCAGGAGAACCAGGAG	^49^
*Csnk2a2*	Casein Kinase 2 Alpha 2	**Forward**: TCCATGGGCAGGACAACTAT **Reverse**: AAAGTTTTCCCAGCGCTTCC	^43^
*Hprt1*	Hypoxanthine Phosphoribosyltransferase 1	**Forward**: GCTGAAGATTTGGAAAAGGTG **Reverse**: AATCCAGCAGGTCAGCAAAG	^47^
*Lama4*	Laminin Subunit Alpha 4	**Forward:** AACTGACCGAGGCTGTCAAG **Reverse**: TGAGGTTTCTCACTGCGTCC	^50^
*Mcam*	Melanoma Cell Adhesion Molecule	**Forward**: GAGGTCACTGTCCCTGTCTT **Reverse**: TCGGTGCTTTCCTCTTCCAT	Designed
*Mtor*	Mechanistic Target of Rapamycin Kinase	**Forward**: AGATACGCCGTCATTCCT **Reverse**: GCTCAAACACCTCCACCT	^51^
*Pecam1*	Platelet Endothelial Cell Adhesion Molecule 1	**Forward**: CTGGGAGGTATCGAATGGGC **Reverse**: CCCGAGACTGAGGAATGACG	^52^
*Rictor*	RPTOR Independent Companion Of MTOR Complex 2	**Forward:** GAGGTGGAGAGGACACAAGCCC **Reverse**: GGCCACAGAACTCGGAAACAAGG	^53^

### Serum-derived extracellular vesicle miRNA and tRNA sequencing

EVs were isolated from serum samples (*n* = 3 per timepoint per treatment (*n* = 6/group); stored at −80℃ until processing) by size exclusion chromatography (SEC) using qEV single 70 nm columns (IZON, Lyon, France) following the manufacturer’s instructions. Total RNA was extracted using the miRNeasy mini kit (Qiagen, Hilden, Germany) and 5 µL total RNA was used as an input for small RNA sequencing library preparation. Next-generation sequencing (NGS) data were analysed using the miND analysis pipeline^54^ with adaptions for tRNA quantification (see **Supplementary methods**). Resulting data are deposited on NCBI GEO, accession GSE293831.

### Statistical analyses

Prior to analyses, qPCR and Bonar scoring data were averaged (per animal per limb). Linear mixed effect models were fitted for each gene or Bonar category using lme4 package using R version 4.4.2 (https://cran.r-project.org/). Effect variables were limb (control vs. injured), treatment (vehicle vs. rapamycin) and timepoint (1 week vs. 3 weeks). For each animal (i.e. subject), repeated measures were accounted for using random effect in the model (i.e. left injured limb vs. right control limb). Normality of the model residuals were assessed via visual inspection of the histogram and Shapiro-Wilk test. Type III Analysis of Variance with Satterthwaite’s method was performed using lmerTest package to obtain p-values for the main effects and interactions in the linear mixed effects models. Post hoc pairwise comparisons (Holm P-value adjustment method) were performed on variables (limb, timepoint and treatment) using phia package. Figures were generated using GraphPad Prism version 10.4.1 for Windows (GraphPad Software, Boston, Massachusetts USA; https://www.graphpad.com/updates/prism-10-4-1-release-notes) and Inkscape version 1.3.2 (https://inkscape.org/release/inkscape-1.3.2/).

## Results

### Rapamycin did not enhance intratendinous healing but limited peritendinous fibrosis at 3 weeks post-injury

**Fig. 2:**
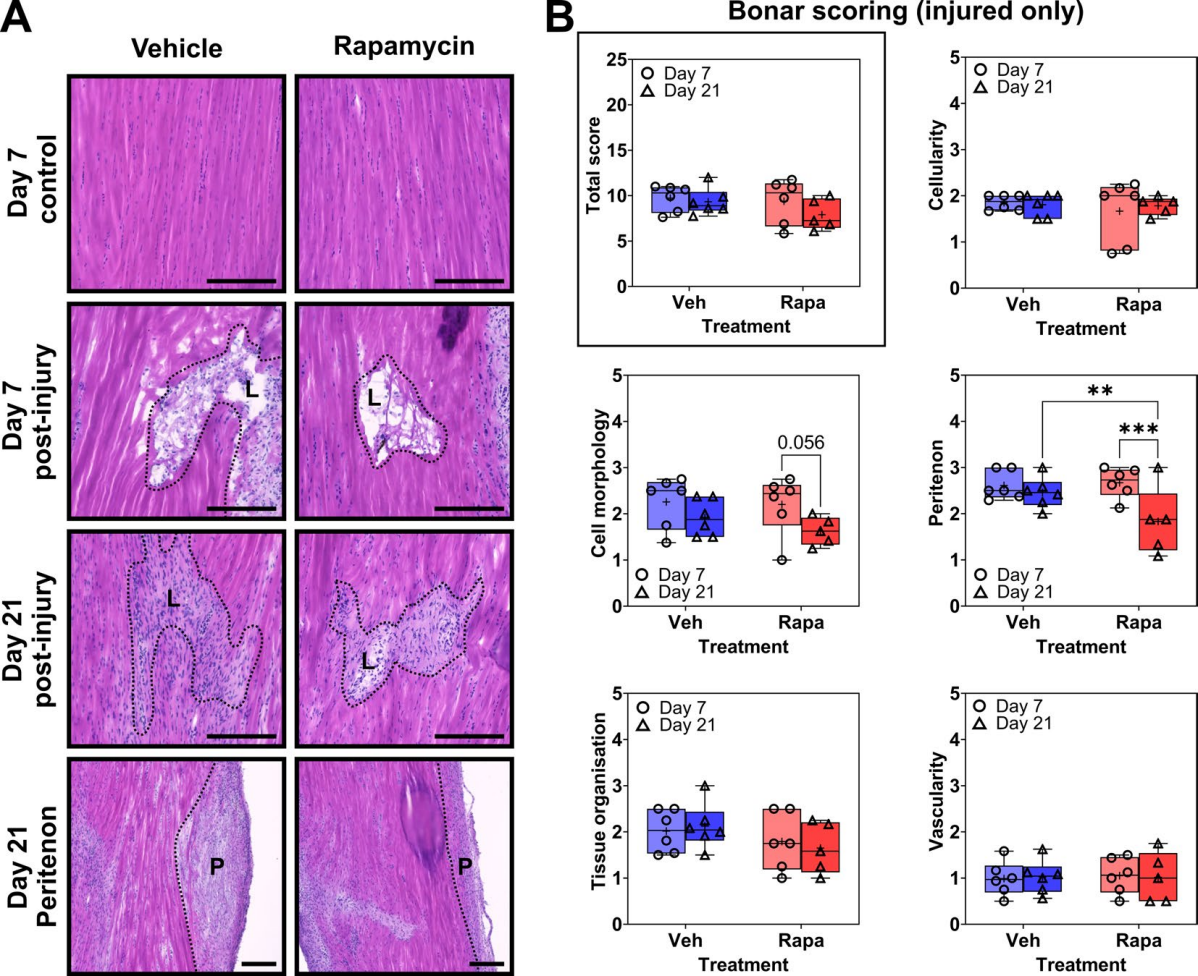
Rapamycin did not improve tendon healing but reduced peritendinous fibrosis. (**A**) Representative images of Achilles tendons at day 7 and day 21 following needle-induced injury and rapamycin treatment. L = lesion. P = peritenon. (**B**) Box-and-Whisker plot featuring minimum and maximum values from Bonar scoring results of independently assessed histological images of tendon injuries, including total score (solid box), cell morphology, cellularity, peritenon fibrosis, tissue organisation and vascularity. are presented. (+) denotes mean. *n* = 6 animals per timepoint per treatment. (*) *P* ≤ 0.05, (**) *P* ≤ 0.01, (***) *P* ≤ 0.001. O = 1 week, △ = 3 weeks. Vehicle = blue, Rapamycin = red.

Tendon injuries were (blindly) assessed histologically at both day 7 and day 21 post-surgery using H&E (Fig. 2A) and subsequently scored using a modified Bonar scoring system (**Table ****S1**). Detailed results, including one- and two-way interactions between variables, are detailed in **Table S2**. Total scores in injured tendons were typically ~ 8–10, readily distinguished from uninjured tendons (total score ~ 3–5) in both treated and untreated groups (**Figure S2**), yet rapamycin treatment had no overall significant effect on post hoc comparisons including total score, cell morphology, cellularity, tissue organisation or vascularity (Fig. 2**)**. Rapamycin treatment did however significantly reduce peritenon scores, with respect to both time and injury: compared to vehicle treatment, rapamycin significantly reduced peritenon scores at day 21 (Fig. 2B; *P* = 0.0038), and these scores were also significantly lower than those at day 7 of rapamycin treatment (Fig. 2B; *P* < 0.001).

**Fig. 3:**
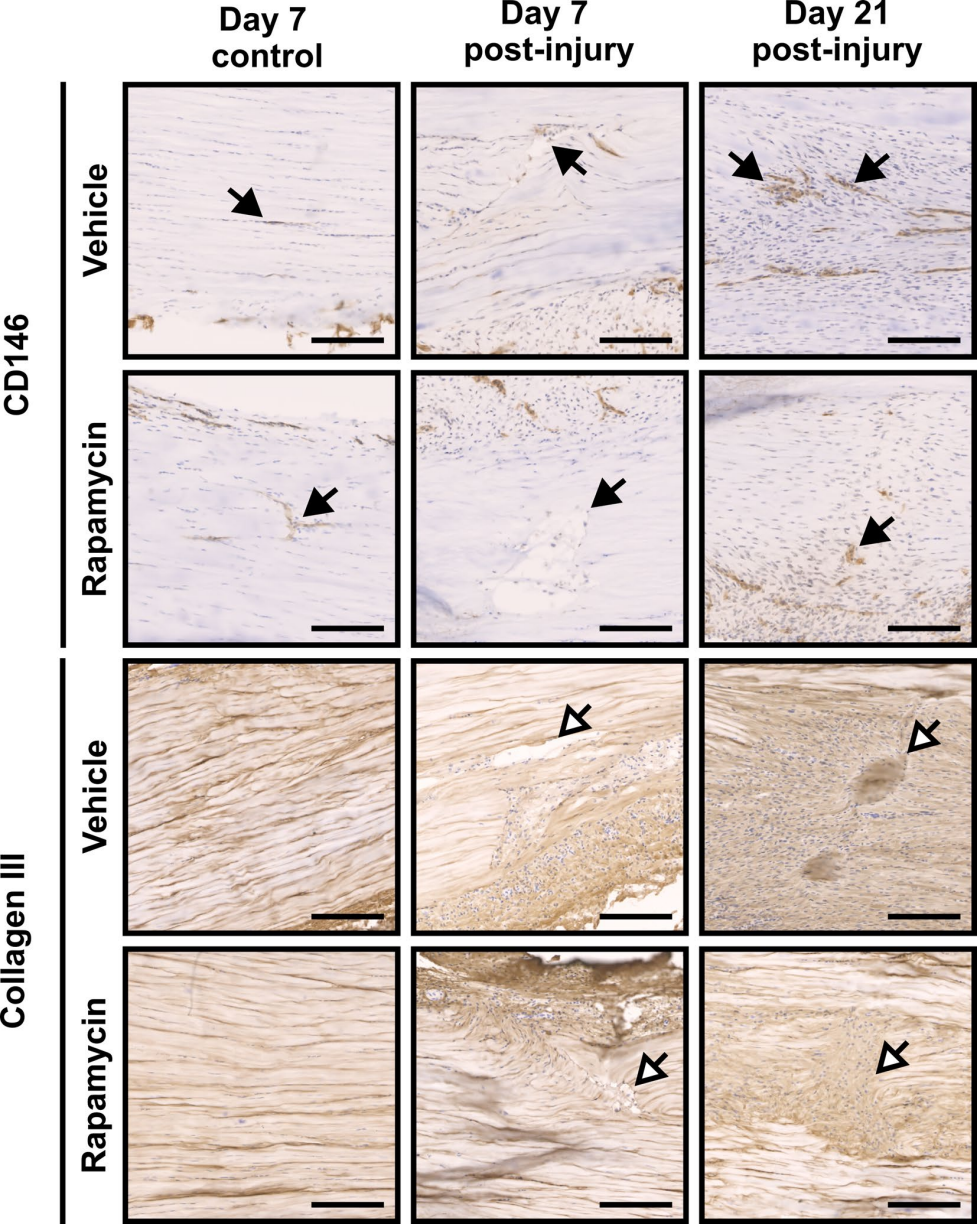
Migration of CD146+ cells into tendon lesions appears reduced with rapamycin treatment whereas deposition of collagen III is unchanged. Immunolabelling for CD146 (top panel) and collagen III (bottom panel) in tendons at days 7 and 21 post injury, with and without rapamycin treatment. Scale bar = 200 μm. Black arrows = CD146+ cells. White arrows = lesion area. *n* = 4 animals per timepoint per treatment.

**Fig. 4:**
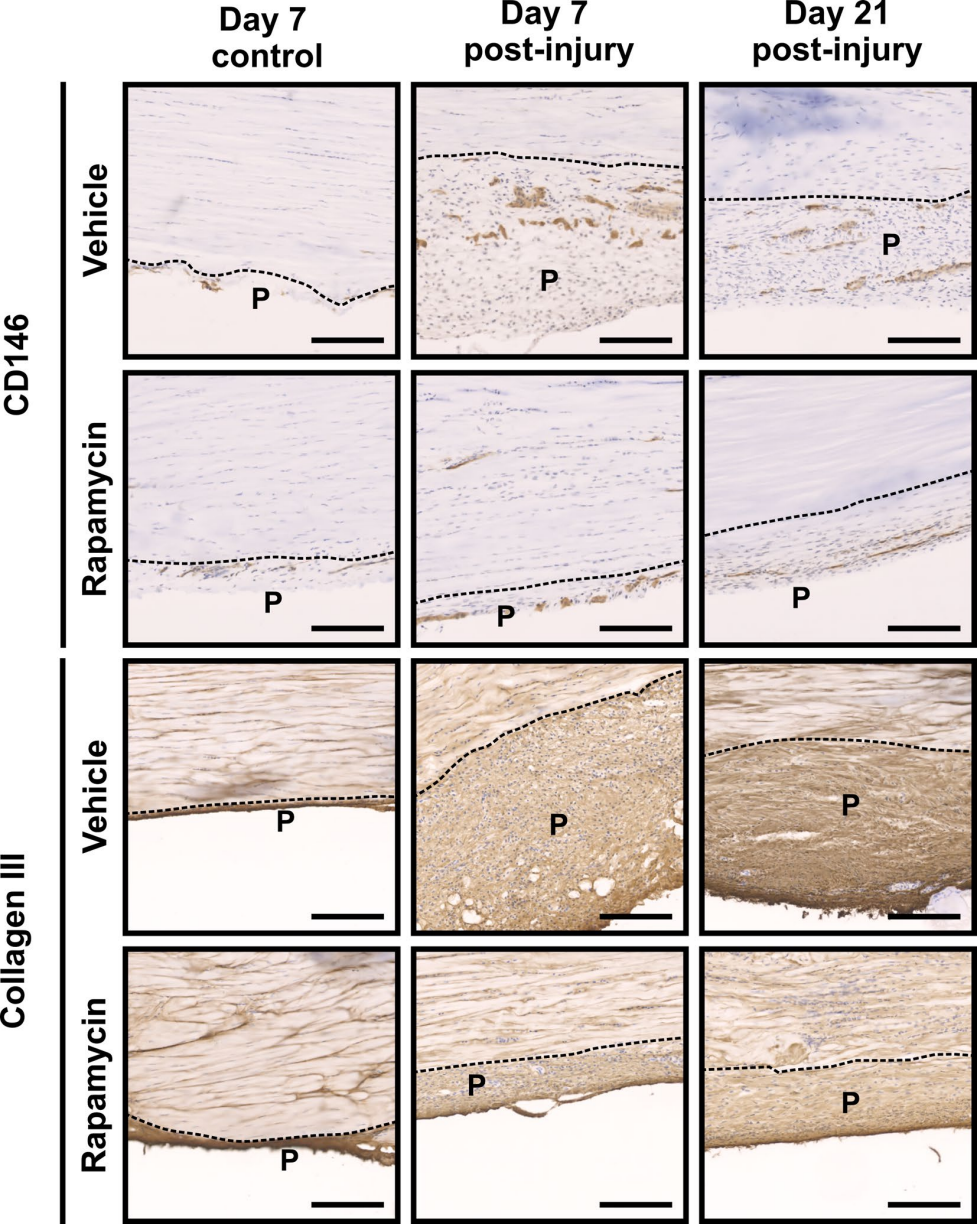
Peritendinous CD146 recruitment and collagen deposition appears diminished by rapamycin treatment. Immunolabelling for CD146 (top panel) and collagen III (bottom panel) in peritendinous regions at days 7 and 21 post injury, with and without rapamycin treatment. Scale bar = 200 μm. P = peritendinous regions (delineated with dashed lines). *n* = 4 animals per timepoint per treatment.

As we have reported previously, CD146+ cells are recruited to tendon injury sites by day 7 post-surgery. We hypothesised that rapamycin treatment would enhance CD146+ cell recruitment and thus improve tendon repair. Accordingly, we used immunolabelling to assess CD146+ cell numbers, and presence of the wound marker collagen type 3 (Col III), both within the lesion and in peritenon regions (Figs. 3 and 4). In uninjured tendons, CD146+ cells were confined to IFM and the peritenon of uninjured tendons, with rapamycin treatment eliciting no apparent difference in CD146+ cell number or distribution. In injured tendons, however, rapamycin treatment resulted in a modest reduction in apparent CD146 labelling at the lesion site as well as in peritendinous regions, at day 7 (but not day 21). Deposition of collagen III within the lesion site was broadly comparable between treatment groups at days 7 and 21 but within the peritenon Col III was markedly reduced by rapamycin treatment (Fig. 4).

### Rapamycin had limited effects on transcriptional circuits associated with tendon injury, mTOR signalling or angiogenesis

**Fig. 5:**
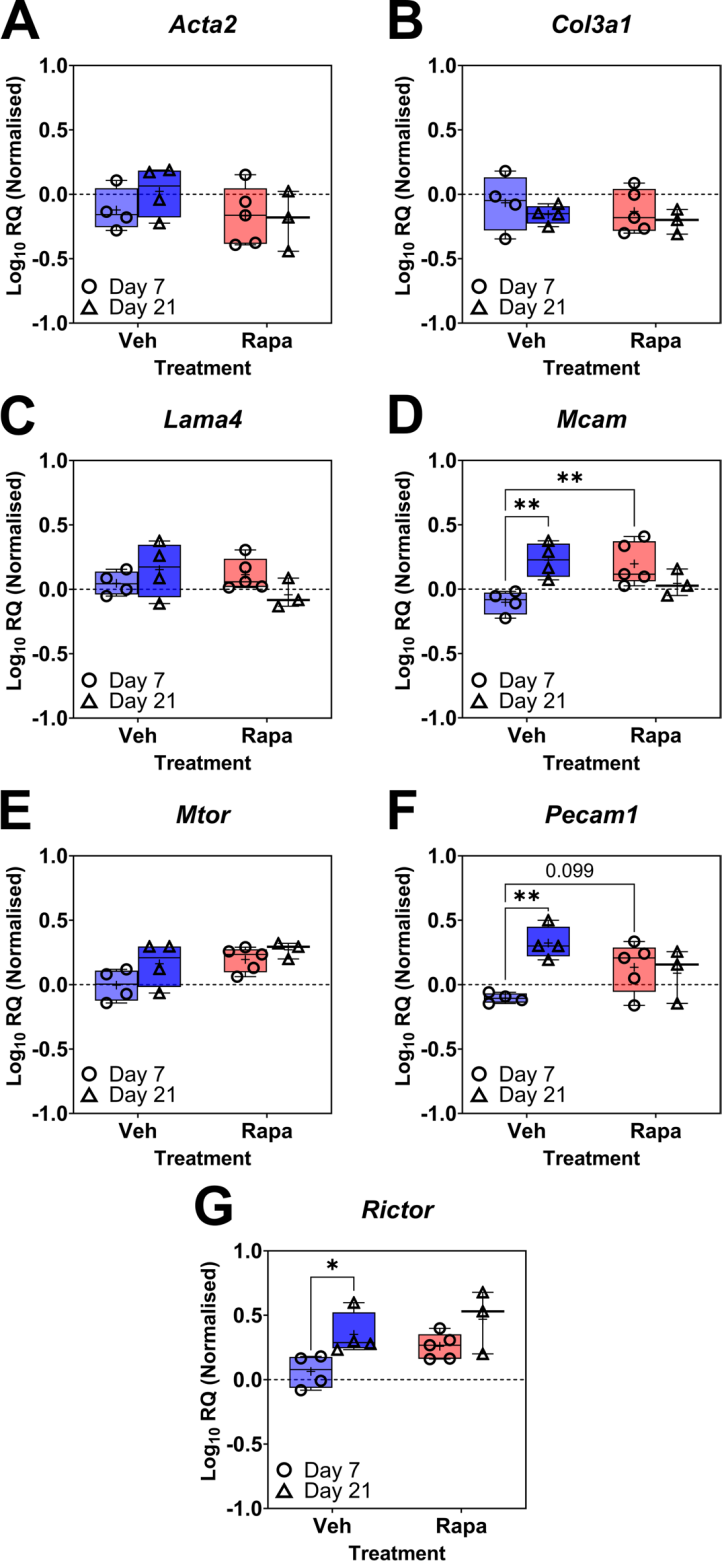
Alterations in gene expression in response to tendon injury and rapamycin treatment. Box-and-Whisker plot featuring minimum and maximum values from qPCR analyses of (**A**) *Acta2*, (**B**) *Col3a1*, (**C**) *Lama4*, (**D**) *Mcam*, (**E**) *Mtor*, (**F**) *Pecam1*, and (**G**) *Rictor* in injured tendons with rapamycin or vehicle treatment at day 7 and day 21 post-surgery. (+) denotes mean. *n* = 3–5 animals per timepoint per treatment: Veh d7 (n) = 4, Veh d21 (n) = 4, Rapa d7 (n) = 5, Rapa d21 (n) = 3. (*) *P* ≤ 0.05, (**) *P* ≤ 0.01, (***) *P* ≤ 0.001. O = 1 week, △ = 3 weeks. Vehicle = blue, Rapamycin = red.

To assess the transcriptional effects of rapamycin treatment on tendon injuries, we measured the expression of genes associated with tendon injury and microdamage (*Acta2* and *Col3a1*), the mTOR pathway (*Rictor*,* Mtor*), angiogenesis (*Pecam1*), as well as the IFM cell population marker CD146 (*Mcam*) and its ligand *Lama4* (Fig. 5); both of which we have previously shown to respond to tendon injury^16^. Detailed results, including one- and two-way interactions between variables, are summarised in **Table S3**. Rapamycin treatment had no significant effect on expression of *Acta2*, *Col3a1*, *Lama4*, *Mtor*, and *Rictor* in injured tendons, but treatment significantly increased *Mcam* expression compared to vehicle alone in injured tendons at day 7 (Fig. 5D; *P* = 0.0034).

### Rapamycin significantly affected circulating miRs primarily at day 7 following tendon injury

Finally, to identify potential systemic biomarkers of tendon injury in response to rapamycin treatment, we isolated, sequenced and analysed serum-derived EV-associated miRs and tRNAs from vehicle and rapamycin treated animals at day 7 and day 21 post-injury (**Figure S4 & Table S4**). It must be noted that no comparisons of uninjured versus injured sera was possible given contralateral uninjured tendons and injured tendons were obtained from the same animals. Of 366 distinct miRs identified, we found five differentially expressed (DE) EV-associated miRs in rapamycin treatment versus vehicle treatment at day 7 (Table 2). The general effect of the treatment was examined by comparing both treatment groups irrespective of timepoints and including the timepoint information as blocking factor, to reduce the intragroup variability. Six DE miRs in rapamycin treated samples compared to vehicle were found, with rno-miR-128-3p overlapping in both analyses (Table 3**).** In addition to miRs, we also explored potential tRNA markers associated with rapamycin treatment. Overall, 420 distinct tRNAs were identified with 391 overlapping across all samples, but no DE tRNAs were reported between treatments.

**Table 2:** Differentially expressed miRs in rapamycin day 7 group versus vehicle day 7 group.

miRNA	Log fold change (logFC)	Log counts per million (logCPM)	*P*-Value	False discovery rate (FDR)
rno-miR-122-5p	1.3	12	0.0008	0.014
rno-miR-191a-5p	−0.47	17	0.0049	0.049
rno-miR-142-3p	−0.9	13	0.0071	0.049
rno-miR-128-3p	−1	15	0.0072	0.049
rno-miR-150-5p	−2.1	13	0.00038	0.013

Entries are sorted by logFC from largest to smallest. For logFC values, logFC > 0 indicates increased expression in rapamycin treatment groups versus vehicle treatment groups. *n* = 3 animals per timepoint per treatment.

**Table 3:** Differentially expressed miRs in rapamycin treatment versus vehicle treatment groups blocking on timepoint).

miRNA	Log fold change (logFC)	Log counts per million (logCPM)		*P*-Value	False discovery rate (FDR)
rno-miR-133a-3p	2		8.9	0.00073	0.026
rno-let-7c-5p	1		12	0.0017	0.045
rno-miR-99a-5p	0.82		13	0.0028	0.05
rno-miR-128-3p	−0.86		15	0.000016	0.0017
rno-let-7f-5p	−1		14	0.000044	0.0024
rno-miR-34a-5p	−1.7		9.3	0.0021	0.046

Entries are sorted by logFC from largest to smallest. For logFC values, logFC > 0 indicated fold change in rapamycin treatment groups versus vehicle treatment groups. *n* = 6 animals per treatment.

## Discussion

In this study, we examined whether daily systemic treatment with rapamycin enhanced tendon healing using an established needle-puncture injury model in the rodent Achilles tendon. In contrast to our hypothesis, our rapamycin regime had a limited effect on tendon core healing as assessed histologically, but instead demonstrated beneficial effects in peritendinous regions. A previous study has reported the anti-fibrotic effects of rapamycin in peritendinous fibrosis affecting rodent flexor tendons, with activation of autophagy provided by rapamycin acting as a protective mechanism against tendon adhesion formation^35^.

In agreement with our previous study^16^, immunolabelling for the IFM cell marker CD146 demonstrated migration of CD146+ cells into the lesion site of injured tendons. This cell recruitment appeared to be reduced by rapamycin, however, with markedly fewer CD146+ cells found in the tendons of treated animals (though we did not quantify cell numbers precisely). Interestingly, studies have shown that CD146 plays a significant role in the stabilisation of both Rictor and mTORC2 activity, driving cell proliferation and migration^55^. In contrast to the apparent reductions at the protein level, at the mRNA level *Mcam* expression was higher in rapamycin-treated injured tendons at day 7. This might indicate independent regulatory programs for CD146 transcription and translation, however we note that RNA was extracted from whole tendon sections (rather than specifically from the lesion site), and thus regional, focal changes in CD146 gene expression might be masked by broader alterations in transcriptional behaviour as a result of rapamycin treatment; that is, *Mcam* gene expression may be enhanced within the peritenon during the acute phases of tendon injury. Alternatively, an upregulation of *Mcam* mRNA under rapamycin treatment may not necessarily reflect changes in CD146+ cell populations but rather an overall enhanced angiogenic transcriptional response. Indeed, previous studies have directly linked increased CD146 mRNA to angiogenesis^57,58^. While our work here primarily focused on known IFM cell markers, canonical markers of tendon development (such as *Scx*, *Tnmd* and *Col1a1*), or of angiogenesis (e.g. *Ang-1*, *Eng*, *Vegf*) might merit investigation in future studies.

One prominent effect of rapamycin treatment in our model was a reduction in size of the peritenon following tendon injury. This peritendinous repair might be elicited by enhanced angiogenesis as suggested above, however, as we show, this repair does not extend to the tendon core. While CD146 has been proposed as a marker of tendon stem/progenitor cells that are able to respond to tendon injury^19^, the direct contribution of CD146+ cells to tendon healing remains to be determined. We speculate it may be part of an angiogenic program involving both distinct CD146 and CD31 cells populations during tendon repair, but further studies are needed to establish both baseline and injury-associated activation of angiogenic potential within the tendon.

Due to the limited regenerative effects of rapamycin reported here, we elected not to assess tendon mechanical properties: any contributions of rapamycin to tendon function, post-injury, consequently remain unknown. Zhang et al^41^. reported limited effects on biomechanical testing with rapamycin treatment, whilst Nie et al^37^. demonstrated that damaging effects of mechanical loading were subdued by rapamycin; the latter was confirmed by whole tendon morphology alone, however, and no biomechanical properties were reported. These studies also evaluated rapamycin treatment over longer terms (8- and 12- weeks respectively), and the limited effects we observed could therefore be a consequence of study length (7 and 21 days). The timing and duration of therapeutic rapamycin regimens remains contentious: longer-term rapamycin treatment (3–12 months) have reported significant increases in longevity and health span^59^, whilst clinical signs of autoimmune disease can be alleviated in sub-45 day regimens^60^.

In addition, early rapamycin treatment in response to skin tissue injury and lymphangioma impedes oedema formation and wound healing, thus use of rapamycin only at a later stage of healing has been recommended for these scenarios^61^. It is plausible that rapamycin treatment has longer term benefits which impact healing at later timepoints due to the acute antilymphangiogenic effects of mTOR inhibition. Rapamycin may also have a larger effect on tendon healing in aged animals: it attenuates age-related changes in tendon mechanical properties in mice and improves tendon-to-bone healing in aged rats^39,40^. Rapamycin could thus be explored as a long-term therapeutic to mitigate age-related injuries to the tendon core and preserve mechanical function. Rapamycin administration may also be of benefit for tendon injuries sustained during development, given the crucial role that mTORC1 has during these earlier stages^62^. Rapamycin may have greater therapeutic potential for tendon disorders in individuals with metabolic diseases, such as hypercholesterolemia (i.e. increased circulating cholesterol levels), which are major contributors towards soft tissue/tendon disease including tendon xanthoma^63–65^. Rapamycin’s effect on angiogenesis could potentially translate as a therapy for tendon health disorders rather than acute injuries; future studies exploring systemic rapamycin treatment to offset poor metabolic health and mitigate soft tissue/tendon disease (in metabolically challenged or obese individuals) might thus be of value. We further note that the single-sex design of our study precludes assessment of sexual dimorphism in response to tendon injury (notably, there is a known sex-dependent response to rapamycin in female rodents, which exhibit increased lifespans and increased blood levels of rapamycin, compared to males, when given equal amounts of rapamycin^66^).

The efficacy of rapamycin delivery can be dose-dependent^67^, though whether this extends to targeting of tendon cell populations is not presently known. Pharmacokinetic measurements of rapamycin levels within tendon and dose-dependent response to rapamycin administration was not assessed in this study, however the fact that systemic delivery of rapamycin resulted in significant increases in *Lama4* and *Mtor* gene expression within uninjured tendons strongly suggests that the systemic delivery and dosage used in this study was capable of eliciting transcriptional changes within tendons. The failure of rapamycin to similarly elicit ameliorative or beneficial changes within injured tendons (neither qualitatively nor quantitatively) consequently indicates that this drug might simply be of limited value in this context.

Finally, we also assessed whether rapamycin treatment altered the serum profile of EV-miRs (or indeed circulatory microRNAs generally); our data suggests that rapamycin had a direct effect on miR profile when compared to vehicle-treated controls, with five DE miRs at day 7. When combining timepoints to compare vehicle and rapamycin groups broadly, we found six DE miRs. Our previous work identified a panel of 16 DE serum miRs associated with tendon injury^16^. In this study, we identified more than 277 overlapping miRs and 400 overlapping tRNAs; indeed, rapamycin treatment was associated with five differentially expressed EV-associated miRs (however, no tRNAs were differentially expressed with rapamycin treatment). Downregulation of miR-128-3p following rapamycin treatment strengthens our hypothesis that this drug alters the angiogenic response to tendon injury, given that miR-128-3p has previously been reported as a modulator of vascular smooth muscle cell phenotype^68^. Of the other miRs identified, few of these miRs have, to the authors’ knowledge, been reported as enriched in either tendon injury or tendon-derived EVs including miR-99a-5p, miR-122-5, miR-133a-3p and miR-150-5p^69–71^. Though we report DE miRs derived from serum EVs, we recognise that SEC alone may not be sufficient for EV isolation. Separation of cell-free serum cargoes, such as EVs, soluble proteins (e.g. albumin) as well as lipid particles, is essential for EV-associated biomarker discovery and target validation. Despite the suitability of SEC for separation of EVs and maintaining their integrity, due to insufficient volumes, we were unable to perform comprehensive characterisation, such as physical properties or biochemical composition, to satisfy recommendations from MISEV guidelines^72^.

In conclusion, we show that a once daily treatment with rapamycin alleviates peritendinous fibrosis post-injury but does not enhance healing of lesions within the tendon core (up to three weeks post-injury). This dosing regime is therefore not an effective therapeutic for acute tendon injury in young adult subjects. Future studies should establish whether alternative rapamycin dosing regimens or delivery strategies are able to improve tendon healing within the tendon core, and explore alternative scenarios where rapamycin might have a more prominent effect, such as aged or metabolically challenged animals.

## Supplementary Information

Below is the link to the electronic supplementary material.

Supplementary Material 1

## Data Availability

The datasets generated and/or analysed during the current study are available in the GEO repository, accession number GSE293831 (URL: https://www.ncbi.nlm.nih.gov/geo/query/acc.cgi? acc=GSE293831).

## References

[1] Kearney, R. S., Parsons, N. & Costa, M. L. Achilles tendinopathy management. *Bone Joint Res.* **2** (10), 227–232 (2013). 24135556 10.1302/2046-3758.210.2000200 PMC3809715

[2] de Jonge, S. et al. Incidence of midportion Achilles tendinopathy in the general population. *Br. J. Sports Med.* **45** (13), 1026–1028 (2011). 21926076 10.1136/bjsports-2011-090342

[3] Kujala, U. M., Sarna, S. & Kaprio, J. Cumulative Incidence of Achilles Tendon Rupture and Tendinopathy in Male Former Elite Athletes. *Clin. J. Sport Med.* **15** (3), 133–135 (2005). 15867554 10.1097/01.jsm.0000165347.55638.23

[4] O’Brien, C., Marr, N. & Thorpe, C. Microdamage in the equine superficial digital flexor tendon. *Equine Vet. J.* **53** (3), 417–430 (2021). 32772396 10.1111/evj.13331

[5] Snedeker, J. G. & Foolen, J. Tendon injury and repair – A perspective on the basic mechanisms of tendon disease and future clinical therapy. *Acta Biomater.* **63**, 18–36 (2017). 28867648 10.1016/j.actbio.2017.08.032

[6] Dyment, N. A. & Galloway, J. L. Regenerative biology of tendon: mechanisms for renewal and repair. *Curr. Mol. Biol. Rep.* **1** (3), 124–131 (2015). 26389023 10.1007/s40610-015-0021-3 PMC4570727

[7] Cook, J. L., Khan, K. M. & Purdam, C. *Achilles tendinopathy Man. Therapy*, **7**(3): 121–130. (2002). 10.1054/math.2002.0458 12372309

[8] Tsai, S. L., Nödl, M. T. & Galloway, J. L. Bringing tendon biology to heel: Leveraging mechanisms of tendon development, healing, and regeneration to advance therapeutic strategies. *Dev. Dyn.* **250** (3), 393–413 (2021). 33169466 10.1002/dvdy.269 PMC8486356

[9] Rich, M. D., Solaiman, R. H. & Hillard, C. *Short-Term Postoperative Complications in Flexor Tendon Repair: A Subcategory Analysis by Surgical Specialty*. *Plast. Surg. (Oakv)*, 22925503241241083. (2024). 10.1177/22925503241241083 PMC11562262 39553516

[10] Gatz, M. et al. Open versus minimally-invasive surgery for Achilles tendon rupture: a meta-analysis study. *Arch. Orthop. Trauma Surg.* **141** (3), 383–401 (2021). 32266518 10.1007/s00402-020-03437-z

[11] Myhrvold, S. B. et al. Nonoperative or Surgical Treatment of Acute Achilles’ Tendon Rupture. *N. Engl. J. Med.* **386** (15), 1409–1420 (2022). 35417636 10.1056/NEJMoa2108447

[12] Patel, D. et al. Structure-function specialisation of the interfascicular matrix in the human achilles tendon. *Acta Biomater.* **131**, 381–390 (2021). 34271169 10.1016/j.actbio.2021.07.019 PMC8388240

[13] Zamboulis, D. E. et al. The Interfascicular Matrix of Energy Storing Tendons Houses Heterogenous Cell Populations Disproportionately Affected by Aging. *Aging Dis.* **15** (1), 295–310 (2024). 37307816 10.14336/AD.2023.0425-1 PMC10796100

[14] Choi, H. et al. Heterogeneity of proteome dynamics between connective tissue phases of adult tendon. *eLife* **9**, e55262 (2020). 32393437 10.7554/eLife.55262 PMC7217697

[15] Marr, N. et al. *The tendon interfascicular basement membrane provides a vascular niche for CD146 + cell subpopulations*. *Front. Cell. Dev. Biology*, 10. (2023). 10.3389/fcell.2022.1094124 PMC9869387 36699014

[16] Marr, N. et al. CD146 Delineates an Interfascicular Cell Sub-Population in Tendon That Is Recruited during Injury through Its Ligand Laminin-α4. *Int. J. Mol. Sci.* **22** (18), 9729 (2021). 34575887 10.3390/ijms22189729 PMC8472220

[17] Flanagan, K. et al. Laminin-411 is a vascular ligand for MCAM and facilitates TH17 cell entry into the CNS. *PLoS One* **7**(7), e40443 (2012). 22792325 10.1371/journal.pone.0040443 PMC3391262

[18] Iorio, V., Troughton, L. D. & Hamill, K. J. Laminins: Roles and Utility in Wound Repair. *Adv. Wound Care (New Rochelle)*. **4** (4), 250–263 (2015). 25945287 10.1089/wound.2014.0533 PMC4397997

[19] Bi, Y. et al. Identification of tendon stem/progenitor cells and the role of the extracellular matrix in their niche. *Nat. Med.* **13** (10), 1219–1227 (2007). 17828274 10.1038/nm1630

[20] Tarafder, S. et al. Tendon stem/progenitor cells regulate inflammation in tendon healing via JNK and STAT3 signaling. *Faseb j.* **31** (9), 3991–3998 (2017). 28533328 10.1096/fj.201700071R PMC5572690

[21] Li, J., Kim, S. G. & Blenis, J. Rapamycin: one drug, many effects. *Cell. Metab.* **19** (3), 373–379 (2014). 24508508 10.1016/j.cmet.2014.01.001 PMC3972801

[22] Zhang, Y. et al. Emerging role of microRNAs in mTOR signaling. *Cell. Mol. Life Sci.* **74**(14), 2613–2625 (2017). 28238105 10.1007/s00018-017-2485-1 PMC5482757

[23] Yokoi, A. & Ochiya, T. Exosomes and extracellular vesicles: Rethinking the essential values in cancer biology. *Semin. Cancer Biol.* **74**, 79–91 (2021). 33798721 10.1016/j.semcancer.2021.03.032

[24] Millar, N. L. et al. MicroRNA29a regulates IL-33-mediated tissue remodelling in tendon disease. *Nat. Commun.* **6**, 6774 (2015). 25857925 10.1038/ncomms7774 PMC4403384

[25] Thankam, F. G. et al. MicroRNAs associated with inflammation in shoulder tendinopathy and glenohumeral arthritis. *Mol. Cell. Biochem.* **437** (1–2), 81–97 (2018). 28634854 10.1007/s11010-017-3097-7 PMC5738295

[26] Yin, H. et al. Lentivirus-mediated overexpression of miR-29a promotes axonal regeneration and functional recovery in experimental spinal cord injury via PI3K/Akt/mTOR pathway. *Neurochem. Res.* **43**(11), 2038–2046 (2018). 30173324 10.1007/s11064-018-2625-5

[27] Li, Y. et al. microRNA-378 promotes autophagy and inhibits apoptosis in skeletal muscle. *Proc. Natl. Acad. Sci. U. S. A.* **115**(46), E10849-e10858 (2018). 30373812 10.1073/pnas.1803377115 PMC6243236

[28] Bardell, D. et al. The role of microRNAs in tendon dysfunction. *Osteoarthr. Cartil.* **26**, S165–S166 (2018).

[29] März, A. M. et al. Large FK506-binding proteins shape the pharmacology of rapamycin. *Mol. Cell. Biol.* **33** (7), 1357–1367 (2013). 23358420 10.1128/MCB.00678-12 PMC3624267

[30] Xie, J., Wang, X. & Proud, C. G. *mTOR inhibitors cancer therapy* **F1000Res**, 5. (2016). 10.12688/f1000research.9207.1 PMC5007757 27635236

[31] Hillel, A. T. & Gelbard, A. Unleashing rapamycin in fibrosis. *Oncotarget* **6** (18), 15722–15723 (2015). 26158293 10.18632/oncotarget.4652 PMC4599220

[32] Molina-Molina, M. et al. Anti-fibrotic effects of pirfenidone and rapamycin in primary IPF fibroblasts and human alveolar epithelial cells. *BMC Pulm. Med.* **18**(1), 63 (2018). 29703175 10.1186/s12890-018-0626-4 PMC5922028

[33] Qureshi, A. T. et al. Inhibition of Mammalian Target of Rapamycin Signaling with Rapamycin Prevents Trauma-Induced Heterotopic Ossification. *Am. J. Pathol.* **187** (11), 2536–2545 (2017). 29029772 10.1016/j.ajpath.2017.07.010 PMC5809339

[34] Yang, G. et al. Rapamycin-induced autophagy activity promotes bone fracture healing in rats Corrigendum in /10.3892/etm.2021.9749. *Exp. Ther. Med.* **10** (4), 1327–1333 (2015). 26622487 10.3892/etm.2015.2660 PMC4577952

[35] Zheng, W. et al. Rapamycin Protects Against Peritendinous Fibrosis Through Activation of Autophagy. *Front. Pharmacol.* **9**, 402 (2018). 29731718 10.3389/fphar.2018.00402 PMC5921906

[36] Nie, D. et al. *Rapamycin Treatment of Tendon Stem/Progenitor Cells Reduces Cellular Senescence by Upregulating Autophagy.* **2021**(1), 6638249 (Stem Cells International, 2021) 10.1155/2021/6638249 PMC7870298 33603790

[37] Nie, D. et al. Mechanical Overloading Induced-Activation of mTOR Signaling in Tendon Stem/Progenitor Cells Contributes to Tendinopathy Development. *Front. Cell. Dev. Biol.* **9**, 687856 (2021). 34322484 10.3389/fcell.2021.687856 PMC8311934

[38] Song, F. C. et al. *High glucose represses the proliferation of tendon fibroblasts by inhibiting autophagy activation in tendon injury*. *Biosci. Rep.*, **42**(3). (2022). 10.1042/BSR20210640 PMC8935382 35293974

[39] Zaseck, L. W., Miller, R. A. & Brooks, S. V. Rapamycin Attenuates Age-associated Changes in Tibialis Anterior Tendon Viscoelastic Properties. *Journals Gerontology: Ser. A*. **71** (7), 858–865 (2016). 10.1093/gerona/glv307 PMC4906327 26809496

[40] Zhang E, Wang X, Zhou Z, Zhang C. Rapamycin-mediated inhibition of the mTOR pathway promotes tendon healing in acollagenase-induced achilles tendinopathy. *J Orthop Surg Res* **21**, 5 (2026) 41318630 10.1186/s13018-025-06524-2 PMC12771740

[41] Chen, G. et al. Mechanical loading modulates heterotopic ossification in calcific tendinopathy through the mTORC1 signaling pathway. *Mol. Med. Rep.* **16** (5), 5901–5907 (2017). 28901376 10.3892/mmr.2017.7380 PMC5865767

[42] Percie du Sert, N. et al. Reporting animal research: Explanation and elaboration for the ARRIVE guidelines 2.0. *PLoS Biol.* **18** (7), e3000411 (2020). 32663221 10.1371/journal.pbio.3000411 PMC7360025

[43] Marr, N. et al. Evaluation of suitable reference genes for qPCR normalisation of gene expression in a Achilles tendon injury model. *PLoS One*. **19** (8), e0306678 (2024). 39190750 10.1371/journal.pone.0306678 PMC11349184

[44] Fearon, A. et al. The Bonar score revisited: region of evaluation significantly influences the standardized assessment of tendon degeneration. *J. Sci. Med. Sport*. **17** (4), 346–350 (2014). 23932935 10.1016/j.jsams.2013.07.008 PMC4856522

[45] Viera, A. J. & Garrett, J. M. Understanding interobserver agreement: the kappa statistic. *Fam Med.* **37** (5), 360–363 (2005). 15883903

[46] Untergasser, A. et al. Primer3—new capabilities and interfaces. *Nucleic Acids Res.* **40** (15), e115–e115 (2012). 22730293 10.1093/nar/gks596 PMC3424584

[47] Lardizábal, M. N. et al. Reference genes for Real-Time PCR quantification of MicroRNAs and messenger RNAs in rat models of hepatotoxicity. *PLoS One* **7**(5), e36323 (2012). 22563491 10.1371/journal.pone.0036323 PMC3341372

[48] Damba, T. et al. Hydrogen sulfide stimulates activation of hepatic stellate cells through increased cellular bio-energetics. *Nitric Oxide*. **92**, 26–33 (2019). 31401106 10.1016/j.niox.2019.08.004

[49] Tsuchiya, Y. et al. Impact of high-intensity interval training on tendon related gene expression in rat Achilles tendon. *Biochem. Biophys. Res. Commun.* **658**, 116–121 (2023). 37030065 10.1016/j.bbrc.2023.03.076

[50] Meléndez, G. C. et al. Non-human Primate and Rat Cardiac Fibroblasts Show Similar Extracellular Matrix-related and Cellular Adhesion Gene Responses to Substance P. *Lung Circulation*. **24** (4), 395–403 (2015). Heart. 10.1016/j.hlc.2014.11.015 PMC4492475 25550118

[51] Zhu, F. et al. *Epigallocatechin Gallate Protects against MNNG-Induced Precancerous Lesions of Gastric Carcinoma in Rats via PI3K/Akt/mTOR Pathway* **2021**(1), 8846813 (Evidence-Based Complementary and Alternative Medicine, 2021). 10.1155/2021/8846813 PMC7880711 33628319

[52] Xing, Y. et al. MicroRNA expression profiles and target prediction in neonatal Wistar rat lungs during the development of bronchopulmonary dysplasia. *Int. J. Mol. Med.* **36** (5), 1253–1263 (2015). 26398774 10.3892/ijmm.2015.2347 PMC4601749

[53] Gambarotta, G. et al. Identification and validation of suitable housekeeping genes for normalizing quantitative Real-Time PCR assays in injured peripheral nerves. *PLoS One* **9**(8), e105601 (2014). 25144298 10.1371/journal.pone.0105601 PMC4140797

[54] Diendorfer, A. et al. *miND (miRNA NGS Discovery pipeline): a small RNA-seq analysis pipeline and report generator for microRNA biomarker discovery studies [version 1; peer review: 2 approved with reservations].* F1000Research, 11(233). (2022). 10.12688/f1000research.94159.2 PMC13173284 42147075

[55] Xu, W. et al. CD146 Regulates Growth Factor-Induced mTORC2 Activity Independent of the PI3K and mTORC1 Pathways. *Cell. Rep.* **29** (5), 1311–1322e5 (2019). 31665642 10.1016/j.celrep.2019.09.047

[56] Halt, K. J. et al. CD146 + cells are essential for kidney vasculature development. *Kidney Int.* **90** (2), 311–324 (2016). 27165833 10.1016/j.kint.2016.02.021

[57] Fürstenberger, G. et al. Real-time PCR of CD146 mRNA in peripheral blood enables the relative quantification of circulating endothelial cells and is an indicator of angiogenesis. *Br. J. Cancer*. **93** (7), 793–798 (2005). 16160694 10.1038/sj.bjc.6602782 PMC2361631

[58] Tu, T. et al. CD146 acts as a novel receptor for netrin-1 in promoting angiogenesis and vascular development. *Cell Res.* **25** (3), 275–287 (2015). 25656845 10.1038/cr.2015.15 PMC4349246

[59] Bitto, A. et al. Transient rapamycin treatment can increase lifespan and healthspan in middle-aged mice. *eLife* **5**, e16351 (2016). 27549339 10.7554/eLife.16351 PMC4996648

[60] Esposito, M. et al. Rapamycin inhibits relapsing experimental autoimmune encephalomyelitis by both effector and regulatory T cells modulation. *J. Neuroimmunol.* **220** (1–2), 52–63 (2010). 20149931 10.1016/j.jneuroim.2010.01.001

[61] Huber, S. et al. Inhibition of the mammalian target of rapamycin impedes lymphangiogenesis. *Kidney Int.* **71** (8), 771–777 (2007). 17299523 10.1038/sj.ki.5002112

[62] Lim, J. et al. mTORC1 Signaling is a Critical Regulator of Postnatal Tendon Development. *Sci. Rep.* **7** (1), 17175 (2017). 29215029 10.1038/s41598-017-17384-0 PMC5719403

[63] Tsouli, S. G. et al. Pathogenesis, detection and treatment of Achilles tendon xanthomas. *Eur. J. Clin. Invest.* **35** (4), 236–244 (2005). 15816992 10.1111/j.1365-2362.2005.01484.x

[64] Waugh, C. M. et al. Mild hypercholesterolemia impacts Achilles sub-tendon mechanical properties in young rats. *BMC Musculoskelet. Disord.* **24**(1), 282 (2023). 37046262 10.1186/s12891-023-06375-0 PMC10091839

[65] Taylor, B., Cheema, A. & Soslowsky, L. Tendon pathology in hypercholesterolemia and Familial Hypercholesterolemia. *Curr. Rheumatol. Rep.* **19**(12), 76 (2017). 29101577 10.1007/s11926-017-0704-2

[66] Miller, R. A. et al. Rapamycin-mediated lifespan increase in mice is dose and sex dependent and metabolically distinct from dietary restriction. *Aging Cell.* **13** (3), 468–477 (2014). 24341993 10.1111/acel.12194 PMC4032600

[67] Foster, D. A. & Toschi, A. Targeting mTOR with rapamycin: One dose does not fit all. *Cell. Cycle*. **8** (7), 1026–1029 (2009). 19270529 10.4161/cc.8.7.8044 PMC2778016

[68] Farina, F. M. et al. miR-128-3p Is a Novel Regulator of Vascular Smooth Muscle Cell Phenotypic Switch and Vascular Diseases. *Circ. Res.* **126** (12), e120–e135 (2020). 32216529 10.1161/CIRCRESAHA.120.316489

[69] Song, K. et al. *Exosomes from tendon derived stem cells promote tendon repair through miR-144-3p-regulated tenocyte proliferation and migration* **13**(1), 80 (Stem Cell Research & Therapy, 2022). 10.1186/s13287-022-02723-4 PMC8867681 35197108

[70] Thankam, F. G. et al. Genes interconnecting AMPK and TREM-1 and associated microRNAs in rotator cuff tendon injury. *Mol. Cell. Biochem.* **454** (1–2), 97–109 (2019). 30306456 10.1007/s11010-018-3456-z PMC6438203

[71] Schanda, J. E. et al. Muscle-Specific Micro-Ribonucleic Acids miR-1-3p, miR-133a-3p, and miR-133b Reflect Muscle Regeneration After Single-Dose Zoledronic Acid Following Rotator Cuff Repair in a Rodent Chronic Defect Model. *Am. J. Sports Med.* **50** (12), 3355–3367 (2022). 36053026 10.1177/03635465221119507

[72] Welsh, J. A. et al. Minimal information for studies of extracellular vesicles (MISEV2023): From basic to advanced approaches. *J. Extracell. Vesicles*. **13** (2), e12404 (2024). 38326288 10.1002/jev2.12404 PMC10850029

