# Supplementary material for: Rapamycin reduces peritendinous fibrosis but has a limited effect on intratendinous healing in a rodent Achilles tendon injury model
Source: Sci Rep. 2026 Mar 26;16:15028. doi: 10.1038/s41598-026-45606-x (PMC13172414; doi:10.1038/s41598-026-45606-x)
Supplement: Supplementary file 1 — Supplementary Material 1 [file 41598_2026_45606_MOESM1_ESM.docx]

**Supplementary methods.**

**Serum-derived extracellular vesicle miRNA and tRNA sequencing.** Sera were stored at -80℃ until processing. EVs were isolated from serum samples (n = 3 per timepoint) by size exclusion chromatography (SEC) using qEV single 70 nm columns (IZON, Lyon, France) following the manufacturer's instructions. Total RNA was extracted using the miRNeasy mini kit (Qiagen, Hilden, Germany) and 5 µL total RNA was used as an input for small RNA sequencing library preparation. To each RNA sample, 1 µL of mind spike-in standards (TAmiRNA, Vienna, Austria) were added using the RealSeq Biofluids library preparation kit (RealSeq Biosciences, US). Adapter-ligated libraries were amplified using barcoded Illumina reverse primers in combination with the Illumina forward primer. Library quality control was performed using DNA1000 Chip (Agilent, United States). An equimolar pool consisting of all sequencing libraries was prepared and sequenced via Nextseq2000 with 100 bp single end reads (Illumina, San Diego, USA).

Next-generation sequencing (NGS) data were analysed using the miND analysis pipeline[1] with adaptions for tRNA quantification. Quality of next-generation sequencing data was evaluated automatically and manually with fastQC v0.12[2] and multiQC v1.14[3]. Data were processed as described previously[4-9]. Briefly, reads were adapter trimmed and quality filtered using cutadapt v3.3[4] and then filtered (minimum length = 17 nucleotides). Mapping steps were performed using bowtie v1.3.0[5] and miRDeep2 v2.0.1.2[6], whereas reads were initially mapped against the genomic reference mRatBN.7.2[7] allowing for two mismatches and subsequently miRBase v22.1[8], filtered for miRNAs of *Rattus norvegicus* only (allowing for one mismatch). For an overview of RNA composition, non-miRNA mapped reads were mapped against RNAcentral v23.0[9] allowing for two mismatches and a maximum of 300 multiple mappings, and then assigned to various RNA species of interest. Read counts for tRNAs are corrected for multiple mapping by dividing the read count by the number of mappings. Adjusted read counts are summarised for isodecoders and isoacceptors. Statistical analysis of pre-processed NGS data was undertaken as described previously[10, 11] using R v4.0.5 and the following packages: pheatmap v1.0.12, pcaMethods v1.82 and genefilter v1.72. Differential expression (DE) analysis was performed with edgeR v3.32[10] using quasi-likelihood negative binomial generalised log-linear model functions. Independent filtering method of DESeq2[11] was adapted for use with edgeR to remove low abundance miRNAs or tRNAs to optimise the false discovery rate (FDR) correction. Resulting data are deposited on NCBI GEO, accession GSE293831.

1. Diendorfer, A., et al., *miND (miRNA NGS Discovery pipeline): a small RNA-seq analysis pipeline and report generator for microRNA biomarker discovery studies [version 1; peer review: 2 approved with reservations].* F1000Research, 2022. **11**(233).

2. Andrews, S., *FastQC: a quality control tool for high throughput sequence data*. 2010, Cambridge, United Kingdom.

3. Ewels, P., et al., *MultiQC: summarize analysis results for multiple tools and samples in a single report.* Bioinformatics, 2016. **32**(19): p. 3047–3048.

4. Martin, M., *Cutadapt removes adapter sequences from high-throughput sequencing reads.* 2011, 2011. **17**(1): p. 3.

5. Langmead, B., et al., *Ultrafast and memory-efficient alignment of short DNA sequences to the human genome.* Genome Biology, 2009. **10**(3): p. R25.

6. Friedländer, M.R., et al., *miRDeep2 accurately identifies known and hundreds of novel microRNA genes in seven animal clades.* Nucleic Acids Research, 2011. **40**(1): p. 37–52.

7. Zerbino, D.R., et al., *Ensembl 2018.* Nucleic Acids Research, 2017. **46**(D1): p. D754–D761.

8. Griffiths‐Jones, S., *The microRNA Registry.* Nucleic Acids Research, 2004. **32**(suppl_1): p. D109–D111.

9. The RNAcentral Consortium, *RNAcentral: a hub of information for non-coding RNA sequences.* Nucleic Acids Research, 2018. **47**(D1): p. D221–D229.

10. Robinson, M.D., D.J. McCarthy, and G.K. Smyth, *edgeR: a Bioconductor package for differential expression analysis of digital gene expression data.* Bioinformatics, 2009. **26**(1): p. 139–140.

11. Love, M.I., W. Huber, and S. Anders, *Moderated estimation of fold change and dispersion for RNA-seq data with DESeq2.* Genome Biology, 2014. **15**(12): p. 550.

**
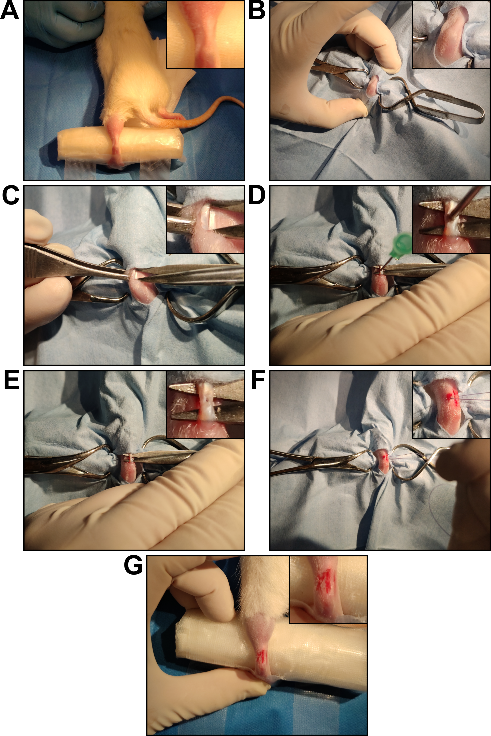
**

**Supplementary figure S1. Tendon injury surgical procedures.** Animals were anaesthetised (isoflurane; 2.5–3%) and pre-operative analgesia was provided (0.05 mg/kg buprenorphine, sub-cutaneous). (A&B) Anaesthetised animals were positioned ventrally, with their left hindlimb extended posteriorly onto a custom-made foot stool to enable tension in the Achilles tendon. (C) The skin over the left Achilles tendon was incised, and the Achilles tendon was isolated from the plantaris tendon and punctured 2 mm proximal to the calcaneal insertion using a 21G hypodermic needle (D&E) by passing the needle through the tendon once. (F&G) Incisions were closed, and all animals were housed in their original groups and behaviour was monitored closely for 48 h using a welfare scoring system. Post-operative analgesia (0.05 mg/kg buprenorphine) was administered subcutaneously twice daily for 48 hours following surgery (i.e. five doses total).

**
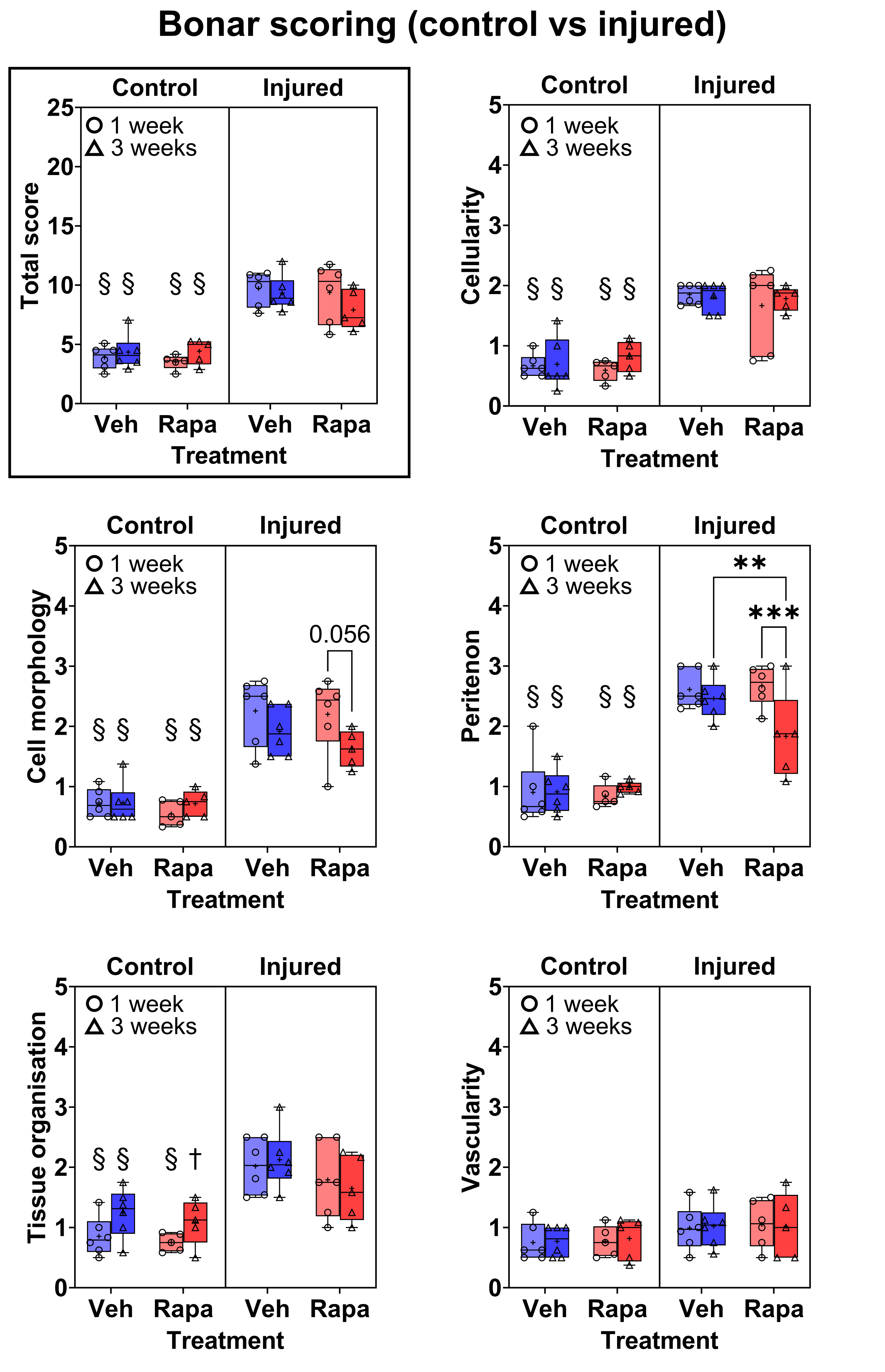
**

**Supplementary figure S2. Complete dataset for BONAR scoring.** (B) Box-and-Whisker plot featuring minimum and maximum values from Bonar scoring results of independently assessed histological images of tendon injuries, including total score (solid box), cell morphology, cellularity, peritenon fibrosis, tissue organisation and vascularity. Note: only statistically significant post-hoc pairwise comparisons between treatment groups (vehicle vs rapamycin) are presented. (+) denotes mean. N=6/group. (*) P≤0.05, (**) P≤0.01, (***) P≤0.001. Symbols denoting significance between control and injured: (†) P≤0.05, (‡) P≤0.01, (§) P≤0.001. O = 1 week, △ = 3 weeks. Vehicle = blue, Rapamycin = red.

**
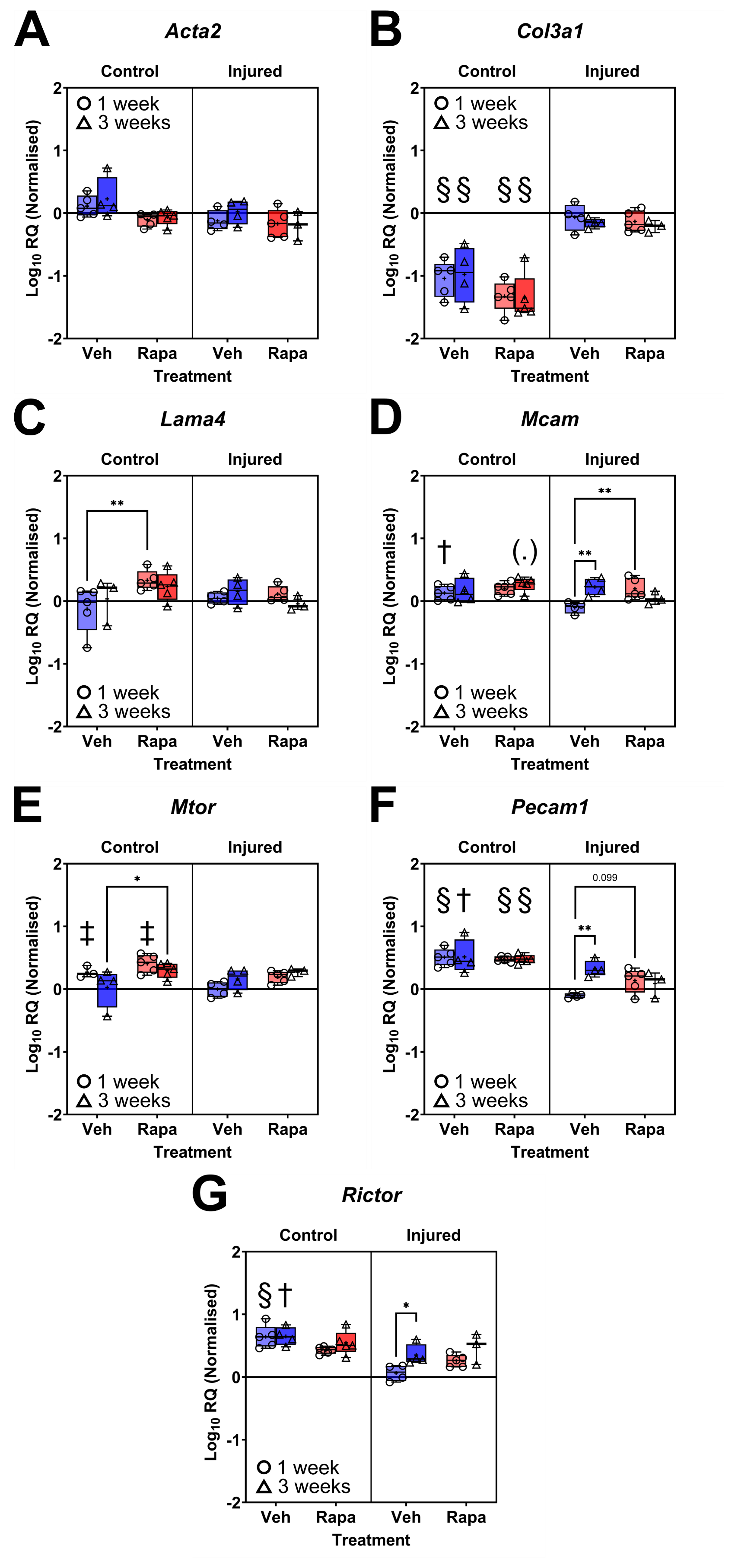
**

**Supplementary figure S3. Complete dataset for qPCR.** Box-and-Whisker plot featuring minimum and maximum values from qPCR analyses of (A) *Acta2*, (B) *Col3a1*, (C) *Lama4*, (D) *Mcam*, (E) *Mtor*, (F) *Pecam1*, and (G) *Rictor* in both uninjured and injured tendons with rapamycin or vehicle treatment at 1 week and 3 weeks post-surgery. Note: only statistically significant post-hoc pairwise comparisons between treatment groups (vehicle vs rapamycin) are presented. (+) denotes mean. N=3-5/group. (*) P≤0.05, (**) P≤0.01, (***) P≤0.001. Symbols denoting significance between control and injured: (.) P≤ 0.1, (†) P≤0.05, (‡) P≤0.01, (§) P≤0.001. O = 1 week, △ = 3 weeks. Vehicle = blue, Rapamycin = red.

**
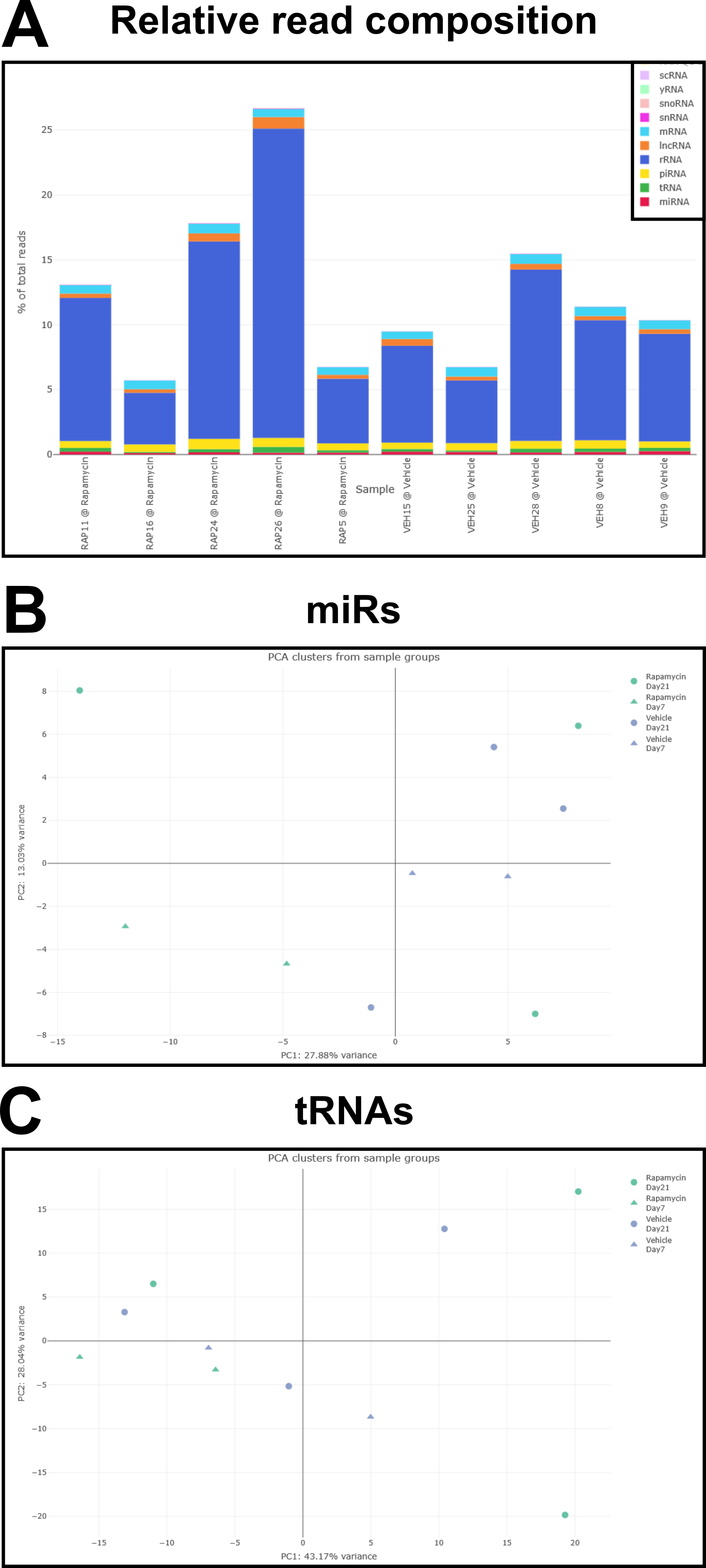
**

**Supplementary figure S4. Serum read composition and principal component analysis (PCA).** (A) relative read composition for all samples carried forward for analyses. Note: reads associated with unclassified reads, quality control and other unknown RNA species are excluded. PCA plots of 10 samples included in (B) miR and (C) tRNA analyses show no apparent clustering.

| Category | | Grade | | | |
| --- | --- | --- | --- | --- | --- |
| Category | | **0** | **1** | **2** | **3** |
| Tendon core | **Cell morphology** | Inconspicuous elongated spindle shaped nuclei with no obvious cytoplasm at light microscopy | Increased roundness: nucleus becomes more ovoid to round in shape without conspicuous cytoplasm | Increased roundness and size; the nucleus is round, slightly enlarged and a small amount of cytoplasm is visible | Nucleus is round, large with abundant cytoplasm and lacuna formation (chondroid change) |
|  | **Cellularity** | Mainly discrete cells | Hypercellular, in rows and/or increased cell numbers | Areas of hypo as well as hyper cellularity | Area of assessment is mostly acellular |
|  | **Vascularity** | No vessels present in FOV/ Inconspicuous blood vessels coursing between bundles | Occasional cluster of vessels | 2–3 clusters of capillaries/vessels | Areas with greater than 3 clusters |
|  | **Tissue Organisation** | Collagen fibres arranged linearly with cell nuclei aligned with long axis of tendon | Small loss of collagen and cell alignment in regions | Moderate loss of collagen and cell alignment | Complete loss of collagen and cell alignment, matrix appears disorganised |
| Peritenon | | Minimal/inconspicuous, 1 cell layer, normal appearance | Enlarged, ~2 cell layers, atypical appearance | Enlarged, presence of distinct vascular bundles | Extensive fibrosis, no organisation, high cellularity |

**Supplementary table 1. Modified Bonar scoring system.**

**Supplementary table 2. ANOVA results BONAR scoring.**

|  | Variable(s) | | | | | | | |
| --- | --- | --- | --- | --- | --- | --- | --- | --- |
| BONAR category | Limb  P-value | Treatment  P-value | Timepoint  P-value | Limb:Treatment  P-value | Limb:Timepoint  P-value | Treatment:Timepoint  P-value | Limb:Treatment:Timepoint  P-value | |
| Cell morphology | ***0.0000(***)*** | 0.12 | 0.10 | 0.49 | ***0.017(*)*** | 0.95 | 0.38 | |
| Cellularity | ***0.0000(***)*** | 0.58 | 0.29 | 0.41 | 0.73 | 0.36 | 0.99 | |
| Peritenon | ***0.0000(***)*** | 0.15 | ***0.047(*)*** | *0.066(.)* | ***0.0018(**)*** | 0.12 | *0.054(.)* | |
| Tissue morphology | ***0.0000(***)*** | 0.17 | 0.27 | 0.19 | 0.15 | 0.68 | 0.72 | |
| Vascularity | ***0.016(*)*** | 0.84 | 0.85 | 0.76 | 0.77 | 0.88 | 0.67 | |
| Total score | ***0.0000(***)*** | 0.21 | 0.80 | 0.20 | ***0.043(*)*** | 0.80 | 0.35 | |
| ^(.) P≤0.1, (*) P≤0.05, (**) P≤0.01, (***) P≤0.001. Significant results are presented in bold, results approaching significance are italicised.^ | | | | | | | |  |

**Supplementary table 3. ANOVA results qPCR.**

|  | Variable | | | | | | | |
| --- | --- | --- | --- | --- | --- | --- | --- | --- |
| Gene | Limb  P-value | Treatment  P-value | Timepoint  P-value | Limb:Treatment  P-value | Limb:Timepoint  P-value | Treatment:Timepoint  P-value | Limb:Treatment:Timepoint  P-value | |
| *Acta2* | **0.020(*)** | **0.017(*)** | 0.38 | 0.34 | 0.77 | 0.45 | 0.78 | |
| *Col3a1* | **0.000(***)** | *0.092(.)* | 0.68 | 0.14 | 0.52 | 0.96 | 0.65 | |
| *Lama4* | 0.52 | 0.13 | 1.00 | **0.023(*)** | 0.70 | 0.16 | 0.99 | |
| *Mcam* | **0.041(*)** | 0.12 | 0.14 | 0.73 | 0.71 | **0.026(*)** | **0.0077(**)** | |
| *Mtor* | **0.047(*)** | **0.0098(**)** | 0.53 | 0.53 | **0.0073(**)** | 0.73 | 0.32 | |
| *Pecam1* | **0.0000(***)** | 0.91 | 0.17 | 0.74 | *0.074(.)* | *0.078(.)* | **0.024(*)** | |
| *Rictor* | **0.0001(***)** | 0.99 | **0.010(*)** | **0.0087(**)** | *0.097(.)* | 0.86 | 0.38 | |
| (.) P≤0.1, (*) P≤0.05, (**) P≤0.01, (***) P≤0.001. Significant results are presented in bold, results approaching significance are italicised | | | | | | | |  |

**Supplementary table 4. Stratification and number of reads mapped to RNA species.**

| sampleID | miRNA | tRNA | piRNA | rRNA | lncRNA | mRNA | snRNA | snoRNA | yRNA | scRNA | RNA QC  spike-in | spike-in calibrator | other RNA species | unclassified genomic | unmapped |
| --- | --- | --- | --- | --- | --- | --- | --- | --- | --- | --- | --- | --- | --- | --- | --- |
| RAP11 | 32745 | 42984 | 77498 | 1616482 | 48261 | 95149 | 2330 | 1362 | 614 | 19 | 15756 | 21207 | 189705 | 3929001 | 8585965 |
| RAP16 | 16448 | 12962 | 85518 | 585906 | 39120 | 98989 | 863 | 338 | 164 | 7 | 17240 | 23264 | 69125 | 4332122 | 9440211 |
| RAP24 | 22770 | 33071 | 105106 | 2015843 | 82708 | 98513 | 3367 | 1033 | 591 | 17 | 11544 | 20164 | 334324 | 3579146 | 6947408 |
| RAP26 | 22435 | 73059 | 112255 | 3854765 | 143675 | 101187 | 5161 | 3886 | 766 | 21 | 13973 | 21393 | 339000 | 3657491 | 7824896 |
| RAP5 | 24064 | 28888 | 85902 | 800896 | 48541 | 95590 | 1208 | 430 | 187 | 7 | 18370 | 23875 | 88218 | 4537207 | 10319952 |
| *Rapamycin (average)* | ***23692*** | ***38193*** | ***93256*** | ***1774778*** | ***72461*** | ***97886*** | ***2586*** | ***1410*** | ***464*** | ***14*** | ***15377*** | ***21981*** | ***204074*** | ***4006993*** | ***8623686*** |
| VEH15 | 32359 | 30541 | 70679 | 1073538 | 74979 | 82103 | 1158 | 616 | 538 | 4 | 15764 | 20720 | 127443 | 3814321 | 9049813 |
| VEH25 | 28999 | 17943 | 82565 | 707128 | 44164 | 105932 | 998 | 395 | 422 | 11 | 19089 | 22254 | 91015 | 4399335 | 9107701 |
| VEH28 | 25278 | 47849 | 96966 | 2147580 | 71057 | 121928 | 2836 | 977 | 429 | 13 | 16083 | 25213 | 207568 | 4439774 | 9065026 |
| VEH8 | 42726 | 55371 | 134007 | 1949402 | 67968 | 149998 | 2734 | 1121 | 589 | 16 | 22807 | 26104 | 360033 | 5977407 | 12289181 |
| VEH9 | 51824 | 50327 | 96631 | 1628519 | 71493 | 134504 | 2121 | 1067 | 400 | 10 | 23666 | 31466 | 188938 | 5615885 | 11740173 |
| *Vehicle (average)* | ***36237*** | ***40406*** | ***96170*** | ***1501233*** | ***65932*** | ***118893*** | ***1969*** | ***835*** | ***476*** | ***11*** | ***19482*** | ***25151*** | ***194999*** | ***4849344*** | ***10250379*** |
